# Using mesenchymal stem cells as a therapy for bone regeneration and repairing

**DOI:** 10.1186/s40659-015-0053-4

**Published:** 2015-11-03

**Authors:** Jin Shao, Weiwei Zhang, Tieyi Yang

**Affiliations:** Department of Orthopaedics, Shanghai Pudong New Area Gongli Hospital, Second Military Medical University, Shanghai, 200135 China; State Key Laboratory of Oncogenes and Related Genes, Renji-Med X Clinical Stem Cell Research Center, Ren Ji Hospital, School of Medicine, Shanghai Jiao Tong University, Shanghai, 200127 China

**Keywords:** Mesenchymal stem cells, Bone regeneration, Bone repairing, Tissue engineering

## Abstract

Bone is a unique tissue which could regenerate completely after injury rather than heal itself with a scar. Compared with other tissues the difference is that, during bone repairing and regeneration, after the inflammatory phase the mesenchymal stem cells (MSCs) are recruited to the injury site and differentiate into either chondroblasts or osteoblasts precursors, leading to bone repairing and regeneration. Besides these two precursors, the MSCs can also differentiate into adipocyte precursors, skeletal muscle precursors and some other mesodermal cells. With this multilineage potentiality, the MSCs are probably used to cure bone injury and other woundings in the near future. Here we will introduce the recent developments in understanding the mechanism of MSCs action in bone regeneration and repairing.

## Introduction

In 1964 Till et al. gave us the idea and definition of stem cell-self-renewal and differentiation [1]. Nowadays clinically it is a common understanding that the regenerative medicine is dependent on the properties of stem cells [2]. Mesenchymal stem cells (MSCs) are thought to be essential for bone-healing because of its ability to differentiate in vitro into both chondrocytes and osteocytes, and provide great potential for tissue engineering.

Tissue engineering is to replace tissues through the usage of specific cell types when the tissue function or structure is lost because of trauma or disease. Usually a 3D scaffold with various growth factors, cytokines, hormones and other biological molecules will be utilized during the engineering, in order to facilitate the generation of a critical mass of specific precursor cells and their differentiation into the required cell type.

As the key process in tissue engineering is to generate enough amount of a certain cell type, stem cells are thought to be the proper starting cells in clinical practice. They could be collected from various tissues, for example bone marrow, adipose, amniotic fluid, umbilical cord blood and even breast milk [3–6]. Among these origins, bone marrow-derived MSCs are the most commonly utilized stem cells for bone repairing, both in research and clinical practice [7]. There are several reports indicating that the transplantation of bone marrow-derived MSCs promotes bone formation at sites of bone defects [8–10]. In this review we will give a brief introduction of MSCs and importance of its usage in tissue repairing.

## Characteristics of MSCs

Self-renewal is a hallmark of all stem cells [1]. The maintenance of self-renewal and pluripotency of stem cells occurs in the stem cell niche, where stem cells are able to receive signals from the stroma or other cells via contact or secreted molecules within this niche [11, 12]. Adult MSCs also share this kind of ability and could differentiate not only into osteoblasts, adipocytes, and chondrocytes, but also into cells of non-mesodermal lineages including hepatocytes, neuron-like cells, and pancreatic cells [13–17].

MSCs could be easily distinguished from other bone marrow cells by their rapid adherence to plastic and their fibroblast-like morphology [18]. For decades cultured plastic-adherent bone marrow cells are the main source of MSCs, but now MSCs could also be derived from other tissues, such as placenta, adipose tissue, peripheral blood, umbilical blood and so on [19–22]. In vitro bone marrow-derived MSCs express the surface markers CD105, CD90 and CD73, but lack CD45, CD34, CD14, CD11b, CD7a and CD19 [23–26]. There are also some MSCs expressing markers, such as CD271 [23]. However, it is lack of evidences showing all the human MSCs have those markers. MSCs derived from different sources also vary in their differentiation capacity. As bone marrow-derived MSCs have better chondrogenic differentiation potential, they are considered to be a proper source for the therapy which facilitates bone repairing and regeneration [27, 28].

Besides the differentiation potential, MSCs also have immune-suppressive activity, which together with their paracrine function plays an essential role in bone regeneration and repairing [28]. This issue will be discussed in the section “Mechanism of MSCs action”.

## Differentiation capacity of MSCs

### Bone marrow-derived MSCs for Bone Engineering

MSCs exist in most of the postnatal tissues, and they all have differentiation capacity and the potential to be utilized in tissue engineering. Theoretically all the MSCs have the ability to differentiate towards osteogenic, chondrogenic and adipogenic lineages. They could be successfully expanded in vitro and induced to different types of subsets when the optimal cultivation protocols were used [29, 30]. As bone marrow-derived MSCs have the strongest chondrogenic differentiation potential, they are thought to be a better choice for bone engineering than the other MSCs sources [27].

### Chondrogenic and osteogenic potential of MSCs in vitro

Even though MSCs with different origins have differences in differentiation capacity, all of them could be derived into osteoblasts and chondrocytes, which are essential in bone repairing. The tendency to differentiate into a certain cell lineage is usually dependent on the culture conditions. Bone marrow-derived MSCs have the highest potential to generate chondrocytes and osteoblasts. And the standard protocol for chondrogenesis was first established for bone marrow-derived MSCs. In this standard protocol growth factors from transforming growth factor β (TGF-β) superfamily are the key factors which facilitate chondrogenic differentiation of MSCs [31, 32]. Previous studies showed that these factors regulate MSCs differentiation via several signaling pathways, such as Smad, extracellular signal-regulated kinase 1/2 and c-Jun N-terminal kinase pathways [33–36].

MSCs derived from other tissues may also be used in clinic because of their advantages. For example, adipose-derived MSCs are easy to obtain, and umbilical cord-derived MSCs have high differentiation capacity and can be stored after birth and used as autologous implantation or tissue engineering. In cultivation with some certain growth factors such as TGF-β and bone morphogenetic protein-6 (BMP-6), adipose-derived MSCs can express cartilage-specific proteins such as type II collagen, but they do not express hypertrophic chondrocyte markers such as type X collagen [37–39]. In 2010 a standard protocol for isolation of adipose-derived MSCs and induction to chondrogenesis was published in Nature, which is very useful in practice [38]. However, in comparison with bone marrow-derived MSCs, the chondrogenic potential of adipose-derived MSCs is quite limited.

Apart from the prominent advantages of umbilical cord-derived MSCs, such as a painless collection procedure and fast self-renewal, they also have a very high differentiation capacity including chondrogenic potential. Not like bone marrow-derived MSCs, umbilical cord-derived MSCs show a gene expression pattern more similar to embryonic stem cells (ESCs), and with proper expansion they could be used as a therapy not only for bone injury but also for other tissues repairing, even for neural injury. The most appreciable advantage of umbilical cord-derived MSCs is that the collection procedure is noninvasive and ethically acceptable [40–42]. Maybe in the future with a proper inducing method, umbilical cord-derived MSCs could be a better choice for bone engineering.

### Influence of oxygen and mechanical stimulation on MSCs differentiation

Besides the culture and growth factors, O_2_ partial pressure and mechanical stimulation are also important factors influencing MSCs differentiation. Usually cells are cultured under normoxic (21 % O_2_) conditions. However, most of the cells in vivo are not under such a high O_2_ concentration. For example, there is no vascular tissue in cartilage, thus the microenvironment in cartilage is hypoxic, where the O_2_ concentration is about 1–5 % [43]. Bone marrow is also under hypoxic condition in vivo and the O_2_ concentration there is about 1–7 % [44]. Though adipose tissue is a vascularized tissue, the O_2_ concentration there is only about 10–15 % [45]. It has been showed that hypoxia promotes chondrogenesis rather than osteogenesis [46–49]. When bone marrow-derived MSCs are expanded under low O_2_ concentration (5 %), the subsequent osteogenesis would be enhanced [50]. In practice, it is a simple way to control the differentiation of MSCs using O_2_. Furthermore, the cell fate can be changed without adding some expensive biological factors.

Mechanical forces, which are generated within the cell in response to its extracellular environment and extrinsic mechanical signals, play a central role in determining MSC fate. Though the exact mechanism of mechanical sensitivity and respond is still not clear, it is believed that integrins and ion channels participate in this mechanotransduction progress [51]. It has been demonstrated that MSCs respond to oscillatory fluid flow with intracellular Ca^2+^ transient increasing, and then increase the proliferation rate, up-regulate osteoblastic gene expression and down-regulate alkaline phosphatase activity [52]. The fact that oscillatory fluid flow could up-regulate the expression of *Runx2, Sox9 and PPARγ* in MSCs, suggested that this kind of mechanical stimuli could regulate gene expression of some transcription factors which participate in MSCs differentiation [53, 54].

## Mechanisms of MSCs action in bone regeneration and repairing

### MSCs contribute to bone regeneration and repairing

With the achievements in the identification, isolation, and cultivation of MSCs, it is not difficult to develop improved technologies for defining therapeutic MSCs and MSC-based bone repairing. In 1998 a study demonstrated that implantation of a bone marrow-derived MSCs supplemented scaffold leaded to bone regeneration in bone fracture. On the other hand, implanted scaffold without MSCs had no such function [55]. Further studies indicated that pre-differentiated osteogenetic MSCs supplemented scaffold had superior healing effects [56, 57]. More interestingly, delivery of MSCs into clinical models of diabetes resulted in higher fracture healing activity than those did not received MSCs [58].

It is believed that MSCs can differentiate into skeletal progenitor cells which generate skeletal tissues in vivo. Transplanted CD45^−^CD146^+^ human bone marrow-derived MSCs have the capacity to generate ectopic bones and hematopoietic microenvironment in vivo [59]. Further studies by Omatsu et al. demonstrated that perivascular MSCs which express CXCL12 were essential for hematopoietic stem cells (HSCs) proliferation [60], and in this way play a role in angiogenesis. As we know, osteogenesis is strongly associated with angiogenesis, it is not difficult to speculate that angiogenesis also contributes to bone regeneration and repairing.

In the early days bone marrow-derived MSCs were thought to achieve their functions via replacing the cells in damaged tissues, but recently it was found that MSCs could also provide paracrine signals to repair vascular injury or modulate pathological immune responses [61]. This issue will be discussed later.

### MSCs homing to the injury site

The way MSCs home to the injury site is still not clear, anyway chemoattractant molecules released at the bone injury site must play an essential role in MSCs attracting. It has been known that MSCs express at least 19 chemokine receptors [62]. Stromal cell-derived factor 1 (SDF-1) expressed by stromal niche is the primary attractant for CXCR4-expressing MSCs and during trauma CXCR4 is up-regulated by MSCs [63, 64]. Many other chemotactic factors such as RANTES, macrophage inflammatory protein-1α (MIP-1α), monocyte chemotactic protein 1 (MCP-1) and so on, also work on MSCs. All these indicate that MSCs homing is attractant/receptor dependent [65]. However, the negative side of the homing property of MSCs is that they may home to other tissues even developing tumors [66, 67] or undergo necrosis/apoptosis, which is very harmful.

### Direct and indirect contributions of MSCs

MSCs display a broad differentiation capacity in vitro, it was originally hypothesized MSCs transplantation would induce tissue repairing by replacing cells in the damaged host tissue. During bone repairing, the progenitor cells will migrate to the injury site and differentiate into osteoblasts and chondrocytes, and then lead to bone remodeling [68, 69].

Despite the long-lasting therapeutic efficacy of MSCs in many in vivo models (such as bone and cartilage repairing, cardiovascular and neurological diseases), the incidence of MSCs engraftment remained surprisingly low [70, 71]. This unexpected low engraftment efficacy implied a major challenge to explain the beneficial effects of the MSCs. Accumulating evidences indicated that the general therapeutic effects of MSCs are due to their ability to modify the host micro-environment rather than their capacity to differentiate and incorporate into the host tissue.

In mice it has been observed that transplanted MSCs migrate to the site of fractures, integrate into the callus and secrete BMP-2 which acts as a disulfide-linked homodimer and induces bone and cartilage formation. It is a candidate as a retinoid mediator, playing a key role in osteoblast differentiation [72]. Adding vascular endothelial growth factor (VEGF) together with BMPs could facilitate both osteogenesis and angiogenesis, leading to higher bone formation activity and mechanical strength [73]. Transplanted osteoblastic progenitors repair the fracture via intramembranous ossification, while less differentiated MSCs lead to endochondral ossification, a process reminiscent of embryonic skeletal development. This indicates that bone repairing function of MSCs is based on its maturation state [74].

It is commonly accepted that osteogenesis is associated with angiogenesis. Studies by De Luca et al. demonstrated that the secretion of angiogenic factors by MSCs could be increased when trauma happened. Under chemokines and hypoxic conditions, TGF-α induces the secretion of a range of growth factors such as VEGF, hepatocyte growth factor (HGF), interleukin 6 (IL-6), IL-8, platelet-derived growth factor subunit B (PDGF-BB), angiopoietin-2 (Ang-2) and so on in bone marrow-derived MSCs via MEK/MAPK and PI3 K/AKT pathways [75]. The angiogenesis process is very complicated involving endothelial cell (EC) survival, proliferation, migration, tube formation and maturation. With the help of MSCs paracrine signal, angiogenesis could be enhanced. In turn this angiogenic function changes the micro-environment of the damaged tissue and then benefits osteogenesis.

The ability to regulate immune response is another important contribution of MSCs during bone repairing. It was reported that MSCs transplantation leads to higher survival rates in graft-versus-host disease than usual [76]. MSCs modulate the inflammatory micro-environment at the trauma site and decrease the levels of IL-1β, IL-6 and tumor necrosis factor-α (TNF-α) [72]. As TNF-α is up-regulated in the injury or fracture sites [77], the function of MSCs results in a limited inflammation reaction which facilitates osteogenesis and angiogenesis and in turn contributes to bone repairing. It is now clear that TNF-α recruits osteoclasts and MSCs to the trauma site and causes apoptosis of hypertrophic chondrocytes in order to facilitate endochondral ossification [78]. Because of the osteoclastic activity, high level of TNF-α can inhibit bone repairing [79]. Thus transplantation of MSCs could regulate exaggerated immune responses and then facilitates fracture repair.

Another study demonstrated that factors released by bone marrow derived MSCs could recruit macrophages and endothelial lineage cells into the wound thus enhancing wound healing [80]. Together, the transplanted MSCs could modify the local micro-environment such as angiogenesis, and suppress exaggerated immune reaction, and therefore lead to bone repairing. Even though the detailed mechanism of the exogenously implanted MSCs therapy is still not clear, its potential is quite promising.

**Fig. 1:**
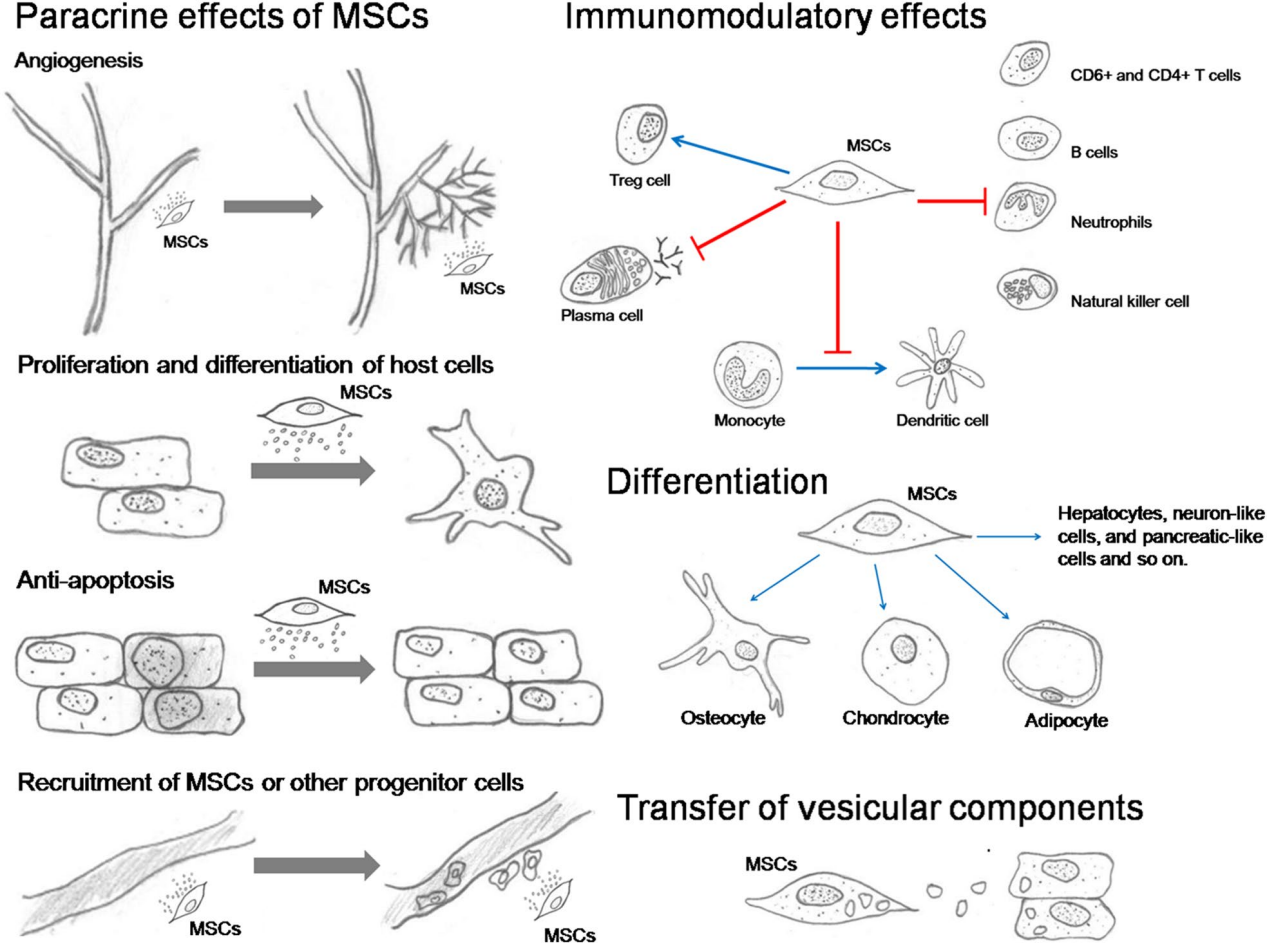
Proposed mechanisms of action of MSCs transplantation. Paracrine effects of MSCs include the stimulation of angiogenesis, protection of other cells from apoptosis, recruitment of host MSCs or other progenitor cells and stimulation of their proliferation and differentiation [82, 83]. The immunomodulatory effects of MSC consist of inhibiting the proliferation and activity of neutrophils, NK cells, B cells, CD4^+^ cells and CD8^+^ T cells, preventing the maturation of monocytes into dendritic cells, suppressing plasma cell immunoglobulin production but stimulating proliferation of regulatory T cells [84]. In some physiological settings, MSCs are able to differentiate into multiple cell types and transfer vesicles containing mRNA, miRNA, proteins and perhaps mitochondria to the host cells [81, 84, 85]

Now there are also evidences indicating that MSCs could possibly transfer mitochondria or vesicular components which contain mRNA, miRNA or proteins [81]. Generally speaking, the basic mechanisms of MSCs action on bone regeneration and repairing are as following (Fig. 1): replacing the damaged cells by proliferation and differentiation; the modulation of the immune system; the secretion of factors that induce tissue repair; recruitment of endogenous MSCs or progenitor cells to the injury site; possible transfer of mitochondria or vesicular components containing mRNA, miRNA and proteins.

## Discussion

Although MSCs were studied because of their differentiation capacity, there is now accumulating evidences suggesting that immunomodulatory and paracrine actions predominantly contribute to their therapeutic efficacy. Some molecules such as VEGF, BMP-2 and MCP-1 play essential roles in the mechanism of MSCs induced tissue regeneration.

Since 1995 more than 3000 patients have received autologous culture-expanded MSCs implantation as a therapy, no serious adverse reaction was reported [86, 87]. However, some unsolved issues still prevent these promising cells to become a realistic treatment option for the future, for example, the absence of a unique marker for MSCs, unstable MSCs isolation and expansion, and recent pre-clinical reports raise the concern that MSCs may cause harmful ectopic bone formation [88]. In order to solve these problems, more preclinical researches about their differentiation capacity and potential effect on the microenvironment need to be done.

In conclusion, over the last decade, numerous studies have been done on MSCs. This is evidenced by the successful application in a wide variety of in vivo wound healing, resulting in an increasing optimism of both basic scientists and clinicians. Besides bone regeneration and repairing, MSCs could be engineered for anti-cancer treatments, designed as carriers for certain factors or genes [89]. Though it is now still impossible to let MSCs become an everyday therapeutic option, routine clinical application of MSCs for a wide variety of pathologies associated with limited angiogenesis, osteogenesis and chondrogenesis is an exciting prospect.

## Abbreviation

BMP-2: bone morphogenetic protein 2
CFU-F: colony-forming unit fibroblast
ESCs: embryonic stem cells
HSCs: hematopoietic stem cells
MSCs: mesenchymal stem cells
PF-4: platelet factor-4
SDF-1: stromal cell-derived factor 1
TGF-α: transformation growth factor α
VEGF: vascular endothelial growth factor
